# Data Augmentation Strength, Training Stability, and Clinical Trade-Offs in Transfer Learning for Chest X-ray Pneumonia Classification

**DOI:** 10.7759/cureus.106519

**Published:** 2026-04-06

**Authors:** Ackley Dias Will

**Affiliations:** 1 Department of Computing, Andrews University, Berrien Springs, USA

**Keywords:** chest x-ray classification, data augmentation, pneumonia, resnet-50, sensitivity, specificity, transfer learning

## Abstract

Background

Transfer learning is widely used in medical imaging when labeled data are limited, but data augmentation policies are often selected empirically, and their interaction with partial fine-tuning is not always evaluated in a controlled manner. This study examined how data augmentation strength influences performance, seed-to-seed stability, and clinically relevant sensitivity-specificity trade-offs in binary pneumonia classification from chest X-ray images using a total of 5,856 chest X-ray images.

Methodology

Using a fixed ResNet-50 backbone, we compared linear probing with a frozen backbone, shallow fine-tuning of the last convolutional block, and deeper fine-tuning of the last two convolutional blocks under the following three augmentation regimes: no augmentation, light augmentation, and strong augmentation. Experiments were repeated across five random seeds and evaluated on a fixed held-out test set using accuracy, F1-score, sensitivity, specificity, area under the receiver operating characteristic curve, and area under the precision-recall curve.

Results

Across augmentation regimes, fine-tuning consistently outperformed linear probing, while differences between fine-tuning conv5 and fine-tuning conv4-5 were small. Relative to no augmentation, light augmentation improved accuracy, F1-score, and specificity while preserving high sensitivity. Strong augmentation yielded the highest mean accuracy and F1-score, but it did not consistently improve the area under the receiver operating characteristic curve or the area under the precision-recall curve relative to light augmentation. Fine-tuned models also showed lower variability across seeds than linear probing.

Conclusions

These findings indicate that augmentation strength is an important determinant of robustness and clinically relevant model behavior, and that augmentation policy should be selected jointly with fine-tuning depth in practical chest X-ray transfer learning pipelines.

## Introduction

Deep learning based on convolutional neural networks is a central component of modern medical imaging systems [1,2]. In chest X-ray analysis, transfer learning from models pretrained on large-scale natural image datasets such as ImageNet is widely used to mitigate limited labeled medical data and can provide strong baselines after task-specific adaptation [3,4]. Related benchmarks and large chest radiograph resources, including CheXpert, CheXNet, ChestX-ray8/14, and MIMIC-CXR, illustrate the established use of deep learning in chest X-ray research [5-8].

Despite broad adoption, transfer-learning design choices remain partly heuristic. One recurring question is how much of a pretrained backbone should be unfrozen for the target task. Prior work in medical imaging suggests that the value of full or partial fine-tuning can depend on the task and data regime, which motivates controlled comparisons of adaptation depth rather than defaulting to a single strategy [9]. A second design choice is data augmentation. Although augmentation is widely used to improve generalization, its practical effect depends on the type, strength, and plausibility of the applied transformations. Image augmentation should therefore be treated as an important modeling choice rather than a routine preprocessing detail [10].

These issues are especially relevant in chest X-ray classification, where performance may vary across datasets and settings and where threshold-dependent clinical behavior can matter as much as threshold-independent ranking metrics [3,4]. In imbalanced binary classification problems, the area under the precision-recall curve can be more informative than the area under the receiver operating characteristic curve alone, and sensitivity and specificity remain essential for understanding false-negative and false-positive behavior at a fixed operating point [11].

A related companion study by our group examined how dataset size influences the relative value of linear probing and partial fine-tuning in the same chest X-ray classification setting [12]. That study varied training-set size and found that larger datasets improved performance across strategies, while deeper unfreezing beyond conv5 provided only modest additional benefit within the evaluated pipeline [12]. The present study addresses a different question. Rather than varying data availability, it keeps the dataset split fixed and isolates the role of data augmentation strength in shaping classification performance, stability across random seeds, and clinically relevant sensitivity-specificity trade-offs.

Accordingly, this work presents a controlled empirical study of the following three transfer-learning strategies within the same ResNet-50-based pipeline: linear probing with a frozen backbone, fine-tuning of conv5, and fine-tuning of conv4-5. Each strategy is evaluated under three augmentation regimes, namely, no augmentation, light augmentation, and strong augmentation. All experiments are repeated across five random seeds, and performance is summarized using mean and standard deviation for aggregate metrics as well as sensitivity and specificity. Rather than proposing a new architecture, this study focuses on how common training design choices jointly influence performance, robustness, and error balance in chest X-ray classification.

## Materials and methods

Dataset

Experiments used the chest X-ray subset of the publicly available pediatric imaging dataset released by Kermany et al., comprising a total of 5,856 chest X-ray images [13]. Only chest X-ray images from the dataset’s predefined chest X-ray directories were included in the study, whereas non-chest X-ray images from the broader source release were excluded. Included labels comprised NORMAL and pneumonia subtypes. Bacterial and viral pneumonia cases were merged into a single PNEUMONIA class to form a binary classification task. No additional manual filtering was performed.

The dataset provides predefined training and test directories [13]. We used the original training directory and created an internal 80/20 split with a fixed random seed to obtain training and validation sets. The predefined test directory was kept fully held out and remained fixed across all experiments. In contrast to our companion dataset-size study, the present analysis kept the data split fixed and varied only the augmentation regime and transfer-learning strategy, allowing the effect of augmentation strength to be examined without changing data availability [12].

Model architecture

**Figure 1:**
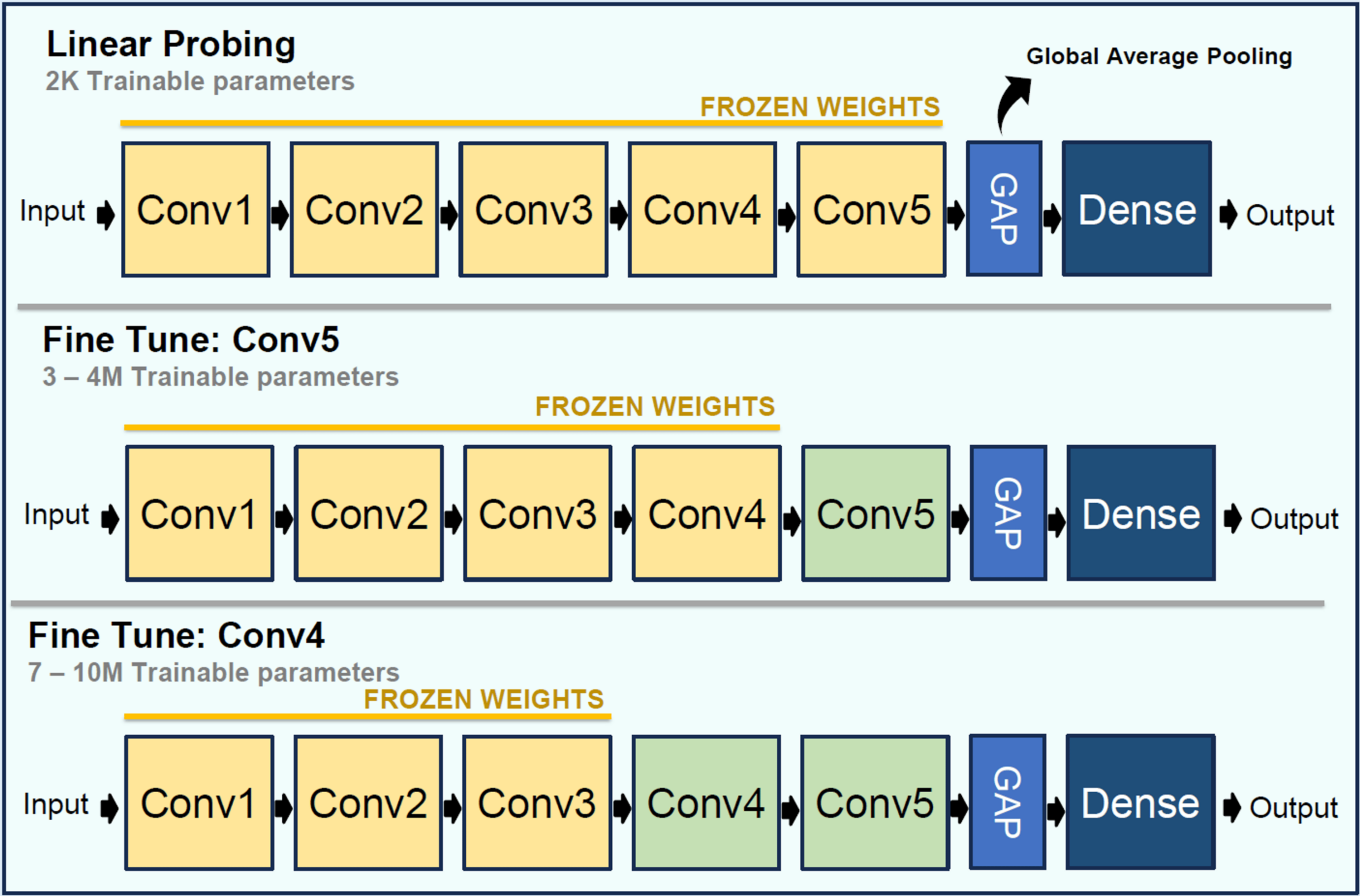
Schematic illustration of the experimental pipeline. The study used a ResNet-50 backbone pretrained on ImageNet and compared linear probing, fine-tuning of conv5, and fine-tuning of conv4-5 under no augmentation, light augmentation, and strong augmentation. Performance was assessed on a fixed held-out test set across five random seeds.

All experiments used ResNet-50 pretrained on ImageNet as the backbone [14,15]. Input images were resized to 224 × 224 and preprocessed using the standard ResNet-50 preprocessing consistent with ImageNet training. A fixed classification head was attached, consisting of global average pooling, a dropout layer with a rate of 0.25, and a sigmoid output neuron for binary classification. No architectural modifications were introduced across experiments. Figure 1 illustrates the overall experimental design and shows how the three transfer-learning strategies were evaluated under the three augmentation regimes within the same ResNet-50-based pipeline.

Three transfer-learning strategies were evaluated. In linear probing, the pretrained backbone was fully frozen, and only the classification head was trained. In shallow fine-tuning, the last convolutional block, conv5, and the classification head were trained jointly, while earlier convolutional blocks remained frozen. In deeper fine-tuning, the last two convolutional blocks, conv4 and conv5, and the classification head were optimized end-to-end, while earlier convolutional blocks remained frozen. These strategies progressively increased trainable capacity while preserving the same backbone and classifier structure across all experiments.

Data augmentation

Three augmentation regimes were compared. In the no-augmentation condition, no data augmentation was applied, and images were passed to the network after resizing and preprocessing only. In the light-augmentation condition, training images were subjected to mild geometric and photometric transformations designed to preserve anatomical structure while introducing limited variability. Specifically, images were randomly rotated within approximately ±3 degrees, zoomed by up to ±10%, and subjected to small contrast perturbations of approximately ±10%; horizontal flipping was also applied during training. In the strong-augmentation condition, training images were subjected to more aggressive but radiographically plausible transformations. These included random rotations within approximately ±7 degrees, horizontal and vertical translations of up to 8% of the image size, asymmetric zooming from approximately -25% to +15%, stronger contrast perturbations of approximately ±25%, and mild Gaussian noise with a sigma of 0.02. A deterministic resize and center crop was applied before random transformations in the strong-augmentation pipeline to introduce scale jitter while preserving image consistency. All augmentation operations were applied only during training, were implemented as part of the model graph using Keras preprocessing layers, and were never applied to validation or test images. The purpose of these regimes was to systematically examine how augmentation strength interacts with model adaptation depth rather than to introduce a novel augmentation method [10].

Training configuration

Optimization used the Adam optimizer [16]. For linear probing, the classification head was trained with a learning rate of 1 × 10^-3^ for up to eight epochs. Training followed a two-phase strategy. In the initial phase, the backbone network was kept frozen when applicable, and only the classification head was trained for eight epochs. In the fine-tuning phase, selected convolutional blocks were unfrozen depending on the experimental configuration. Fine-tuning was performed for 12 epochs when unfreezing conv5 and for 15 epochs when unfreezing conv4-5. Lower learning rates were used during fine-tuning to preserve pretrained representations, with conv5 fine-tuning using 1 × 10^-5^ and conv4-5 fine-tuning using 5 × 10^-6^.

Early stopping was used to prevent overfitting, monitoring the validation area under the precision-recall curve with a patience of eight epochs and restoring the best model weights. ReduceLROnPlateau reduced the learning rate by a factor of 0.5 when the validation area under the precision-recall curve did not improve for three consecutive epochs, with a minimum learning rate of 1 × 10^-6^. Model checkpoints were saved based on the best validation area under the precision-recall curve. The batch size was fixed at 32 for all experiments.

Experiments were implemented in TensorFlow 2.10.1. Training was performed on a system with one NVIDIA GeForce RTX 5070 Ti GPU and a 13th Gen Intel(R) Core(TM) i7-13700 CPU. Input preprocessing used the standard ResNet-50 preprocessing consistent with ImageNet training. Area under the precision-recall curve was computed using tf.keras.metrics.AUC(curve="PR", name="auprc").

Class imbalance handling

To address class imbalance, class-weighted loss was applied during training using inverse class frequency computed from the training subset. N denoted the total number of training samples, nc denoted the number of training samples belonging to class c, and c ∈ {0,1} corresponded to the NORMAL and PNEUMONIA classes, respectively. Class weights were computed as w_c = N / (2n_c). In the full training subset, the class counts were 1,086 NORMAL images and 3,100 PNEUMONIA images. Binary cross-entropy was used as the loss function.

Multi-seed evaluation protocol

To account for training stochasticity, each experiment was repeated using five random seeds: 0, 10, 20, 30, and 40. All reported results correspond to aggregated statistics across these independent runs. Final evaluation was performed on the held-out test set, which was never used during training or model selection.

Evaluation metrics

Performance was assessed using accuracy, F1-score, sensitivity, specificity, area under the receiver operating characteristic curve, and area under the precision-recall curve. Because the dataset is imbalanced toward the PNEUMONIA class, threshold-independent ranking metrics were reported together with threshold-dependent clinical metrics. Accuracy was included as a standard summary metric, but it was not interpreted in isolation. Sensitivity was defined as true positives divided by true positives plus false negatives and reflects the ability to identify pneumonia cases correctly. Specificity was defined as true negatives divided by true negatives plus false positives and reflects the ability to identify normal cases correctly. The F1-score was included as the harmonic mean of precision and recall. The area under the precision-recall curve was reported because it is often more informative than the area under the receiver operating characteristic curve in imbalanced binary classification settings [11]. Unless otherwise stated, all threshold-dependent metrics were computed using a fixed decision threshold of 0.5 for the predicted PNEUMONIA probability.

## Results

Overall performance across augmentation regimes and transfer-learning strategies

Across all augmentation regimes, fine-tuning consistently outperformed linear probing on the held-out test set. Linear probing achieved reasonable sensitivity but lower accuracy and substantially reduced specificity. In contrast, both fine-tuning strategies produced higher and more stable performance across the reported metrics. Differences between fine-tuning conv5 and fine-tuning conv4-5 were small across accuracy, F1-score, area under the receiver operating characteristic curve, and area under the precision-recall curve, indicating limited additional benefit from deeper unfreezing in this setting. Data augmentation also had a clear effect on performance. Relative to no augmentation, light augmentation improved accuracy, F1-score, and specificity while preserving high sensitivity. Strong augmentation yielded the highest mean accuracy and F1-score overall, but it did not consistently improve the area under the receiver operating characteristic curve or the area under the precision-recall curve relative to light augmentation. The overall ranking-based metrics are summarized in Table 1, whereas the threshold-dependent classification metrics are summarized in Table 2.

**Table 1:** Mean ± standard deviation of accuracy, area under the receiver operating characteristic curve (ROC-AUC), and area under the precision-recall curve (AUPRC) across augmentation regimes and transfer-learning strategies.

DA regime	Model	Accuracy	ROC-AUC	AUPRC
NoDA	FT conv4-5	0.9076922 ± 0.002430081	0.9823998 ± 0.001056511	0.9897866 ± 0.000624578
NoDA	FT conv5	0.9060898 ± 0.001827219	0.9823978 ± 0.001063471	0.9897840 ± 0.000624586
NoDA	LP	0.8955128 ± 0.009708186	0.9692296 ± 0.007921743	0.9827830 ± 0.004183661
Light	FT conv4-5	0.9291666 ± 0.007635643	0.9792068 ± 0.001181000	0.9868960 ± 0.000982798
Light	FT conv5	0.9285256 ± 0.008296458	0.9791848 ± 0.001186601	0.9868840 ± 0.000985601
Light	LP	0.8810896 ± 0.030938037	0.9643590 ± 0.002932688	0.9800850 ± 0.001560356
Strong	FT conv4-5	0.9333332 ± 0.002430411	0.9763138 ± 0.002039987	0.9855914 ± 0.001683538
Strong	FT conv5	0.9326922 ± 0.003399416	0.9763160 ± 0.002047668	0.9855934 ± 0.001691031
Strong	LP	0.8842948 ± 0.026488171	0.9599494 ± 0.002224946	0.9763276 ± 0.002651820

**Table 2:** Mean ± standard deviation of F1-score, sensitivity, and specificity across augmentation regimes and transfer-learning strategies.

DA regime	Model	F1-score	Sensitivity	Specificity
NoDA	FT conv4-5	0.9299964 ± 0.001770088	0.9810254 ± 0.002293311	0.7854702 ± 0.006338692
NoDA	FT conv5	0.9287262 ± 0.001450980	0.9789742 ± 0.004931952	0.7846154 ± 0.007763143
NoDA	LP	0.9191206 ± 0.007120325	0.9497436 ± 0.026123575	0.8051280 ± 0.052652507
Light	FT conv4-5	0.9441986 ± 0.005708697	0.9584616 ± 0.008956125	0.8803418 ± 0.024362962
Light	FT conv5	0.9437326 ± 0.006112018	0.9584616 ± 0.008956125	0.8786322 ± 0.027629549
Light	LP	0.9097172 ± 0.020085477	0.9512822 ± 0.017200655	0.7641024 ± 0.109804696
Strong	FT conv4-5	0.9476890 ± 0.001800222	0.9661542 ± 0.002145196	0.8786324 ± 0.007763418
Strong	FT conv5	0.9471898 ± 0.002477726	0.9656414 ± 0.002293311	0.8777778 ± 0.011144136
Strong	LP	0.9098346 ± 0.016801847	0.9287180 ± 0.038776372	0.8102562 ± 0.119030693

Seed-to-seed stability

The multi-seed evaluation showed that performance variability was influenced by both the training strategy and the augmentation regime. Linear probing exhibited the highest variance, particularly in specificity, indicating sensitivity to initialization and the limited adaptability of frozen representations. Fine-tuned models demonstrated substantially lower variance across all metrics. This contrast was most pronounced under light and strong augmentation, where linear probing showed markedly higher variability while fine-tuned models remained consistently stable. For fine-tuned configurations, standard deviations for accuracy and F1-score remained below 1% across all augmentation regimes. These results indicate that combining fine-tuning with data augmentation produced more reliable and reproducible models in this experimental setting.

Sensitivity-specificity trade-offs

**Figure 2:**
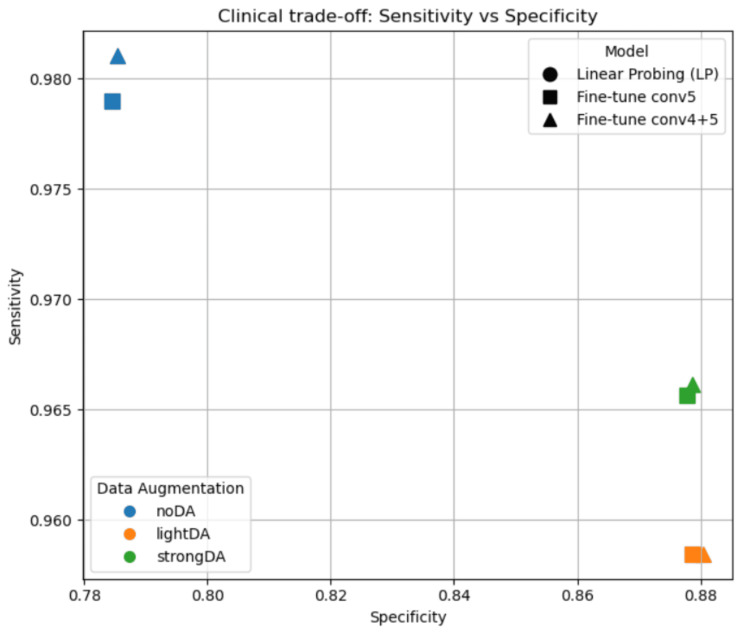
Clinical trade-off between sensitivity and specificity across data augmentation regimes and transfer-learning strategies. Colors indicate the data augmentation regime, while marker shapes indicate the transfer-learning strategy. Each point represents the mean performance across random seeds.

Clinically relevant differences emerged when comparing sensitivity and specificity across model configurations. All fine-tuned models achieved high sensitivity, reflecting strong pneumonia detection performance. However, specificity varied substantially depending on the augmentation regime. Models trained without data augmentation favored sensitivity at the expense of specificity, resulting in higher false-positive rates. Among fine-tuned models, light and strong augmentation improved specificity while preserving high sensitivity. Among fine-tuned models, fine-tuning conv5 and fine-tuning conv4-5 occupied nearly identical regions in the sensitivity-specificity space. These results were obtained using a fixed decision threshold of 0.5, which indicates that the observed trade-offs arose from training design choices rather than post hoc threshold tuning. As shown in Figure 2, the no-augmentation setting tended to favor sensitivity at the expense of specificity, whereas augmented training produced a more balanced operating point.

Confusion matrix analysis

**Figure 3:**
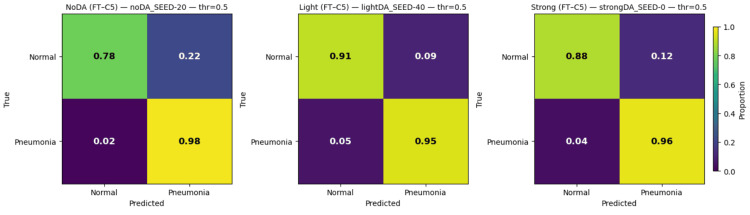
Normalized confusion matrices for the fine-tuning conv5 strategy under no augmentation, light augmentation, and strong augmentation. For each regime, a representative run was selected as the seed whose F1-score was closest to the median across all runs. Matrices are row-normalized per true class and computed on the held-out test set using a fixed decision threshold of 0.5.

Normalized confusion matrices further illustrated the effect of data augmentation on error distribution. For the fine-tuning conv5 strategy, models trained without augmentation showed a higher proportion of false positives, consistent with their lower specificity. In contrast, both light and strong augmentation reduced false-positive rates while maintaining high true-positive rates, leading to more balanced classification outcomes. Differences between light and strong augmentation were subtle, suggesting that moderate transformations may be sufficient to improve robustness without introducing excessive variability. These class-specific error patterns are shown in Figure 3.

## Discussion

This study systematically examined how augmentation strength influences transfer-learning behavior in chest X-ray pneumonia classification under a fixed dataset split and a fixed backbone architecture. The principal finding is that augmentation strength materially affected both average performance and clinically relevant error balance, whereas deeper unfreezing beyond conv5 provided only limited additional benefit in the evaluated setting. Fine-tuning consistently outperformed linear probing across all augmentation regimes, but fine-tuning conv5 and fine-tuning conv4-5 achieved very similar results across the reported metrics. The aggregate metric patterns presented in Table 1 and Table 2, together with the sensitivity-specificity relationships shown in Figure 2, support this interpretation.

Data augmentation should therefore not be treated as a minor implementation detail. Relative to no augmentation, light augmentation improved accuracy, F1-score, and specificity while preserving high sensitivity. Strong augmentation yielded the highest mean accuracy and F1-score, but it did not consistently improve the area under the receiver operating characteristic curve or the area under the precision-recall curve relative to light augmentation. Within the present experimental setting, these findings suggest that increasingly aggressive augmentation does not guarantee uniformly better ranking behavior and may offer diminishing returns compared with moderate augmentation [10]. The confusion matrix patterns in Figure 3 further support this conclusion by showing that augmentation reduced false-positive predictions relative to the no-augmentation condition.

The multi-seed design also adds an important reproducibility perspective. Fine-tuned models were not only stronger on average but also more stable across repeated runs, whereas linear probing showed substantially greater variability, particularly in specificity. In practical terms, a model configuration that is slightly simpler but much more consistent across runs may be more useful than one that appears competitive in a single run but is less stable.

The present findings also reinforce the importance of reporting more than a single summary metric. In imbalanced classification settings, the area under the receiver operating characteristic curve and the area under the precision-recall curve describe ranking quality across thresholds, whereas sensitivity and specificity describe performance at a selected operating point [11]. In this study, augmentation strength affected the balance between sensitivity and specificity even when differences in ranking-based metrics were modest. This is especially relevant in screening-oriented applications, where excessive false positives may increase downstream review burden.

This study is intended to complement, not replicate, our recently published companion study on dataset size and fine-tuning depth [12]. The companion article varied training-set size to determine how the relative benefit of linear probing, fine-tuning conv5, and fine-tuning conv4-5 changes across data regimes [12]. By contrast, the present study keeps data availability fixed and focuses on how augmentation strength affects performance, seed-to-seed stability, and clinically relevant sensitivity-specificity trade-offs. That distinction is central to the present study’s contribution.

From a practical standpoint, the results favor fine-tuning conv5 as a strong compromise between adaptability and complexity. In the present study, it captured most of the gains of deeper unfreezing while avoiding the need to optimize a larger fraction of the backbone. At the same time, the data suggest that moderate augmentation provided a favorable sensitivity-specificity balance, while strong augmentation yielded the highest mean accuracy and F1-score.

This study has several limitations. First, as it evaluated a single backbone architecture, ResNet-50, the observed trends may not transfer directly to other pretrained models. Second, the analysis was restricted to one publicly available pediatric chest X-ray dataset and a binary NORMAL-versus-PNEUMONIA task, which may limit generalizability to other populations, label structures, or thoracic imaging tasks. Third, the augmentation regimes were manually defined rather than learned or adaptively optimized. Fourth, the results reflect repeated runs on a fixed held-out split rather than external validation across institutions. Accordingly, the findings should be interpreted as controlled empirical guidance within the present setting rather than as a universally optimal prescription for transfer-learning practice. The study reports descriptive comparisons of means and standard deviations across seeds rather than formal significance testing. Accordingly, statements regarding relative benefit should be interpreted qualitatively within the present controlled setting.

## Conclusions

In this controlled study of transfer learning for chest X-ray pneumonia classification, augmentation strength had a meaningful effect on model robustness and clinically relevant classification behavior. Relative to no augmentation, light augmentation improved accuracy, F1-score, and specificity while preserving high sensitivity, and strong augmentation produced the highest mean accuracy and F1-score without consistently improving the area under the receiver operating characteristic curve or the area under the precision-recall curve over light augmentation. Across all augmentation regimes, fine-tuning outperformed linear probing, whereas deeper unfreezing beyond conv5 provided only limited additional benefit. These findings support selecting augmentation policy jointly with fine-tuning depth rather than treating augmentation as an isolated preprocessing decision. In the present setting, moderate augmentation provided a favorable sensitivity-specificity balance, while fine-tuning conv5 achieved performance comparable to deeper unfreezing with lower training complexity.
